# Lyme Neuroborreliosis in Children

**DOI:** 10.3390/brainsci11060758

**Published:** 2021-06-07

**Authors:** Sylwia Kozak, Konrad Kaminiów, Katarzyna Kozak, Justyna Paprocka

**Affiliations:** 1 Students’ Scientific Society, Department of Pediatric Neurology, Faculty of Medical Sciences in Katowice, Medical University of Silesia, 40-752 Katowice, Poland; sylwiakozak@icloud.com (S.K.); katarzynakozak@icloud.com (K.K.); 2 Department of Pediatric Neurology, Faculty of Medical Sciences in Katowice, Medical University of Silesia, 40-752 Katowice, Poland

**Keywords:** lyme borreliosis, neuroborreliosis, children, meningitis, facial nerve palsy, neurological manifestations, treatment, diagnosis

## Abstract

Lyme neuroborreliosis (LNB) is an infectious disease, developing after a tick bite and the dissemination of *Borrelia burgdorferi sensu lato* spirochetes reach the nervous system. The infection occurs in children and adults but with different clinical courses. Adults complain of radicular pain and paresis, while among the pediatric population, the most common manifestations of LNB are facial nerve palsy and/or subacute meningitis. Moreover, atypical symptoms, such as fatigue, loss of appetite, or mood changes, may also occur. The awareness of the various clinical features existence presented by children with LNB suspicion remains to be of the greatest importance to diagnose and manage the disease.

## 1. Introduction

Lyme borreliosis (LB) is a tick-borne disease caused by spirochetes of the *Borrelia burgdorferi sensu lato complex,* especially by the following genospecies: *B. burgdorferi sensu stricto, B. afzelii*, and *B. garinii* [1]. The transmission occurs after the bite of the Ixodes tick (*Ixodes* *ricinus* in Europe, *Ixodes scapularis* in the U.S.A., and the other species in different geographical locations), which introduces the invasive spirochetes into the bloodstream. As *Borrelia* pathogens show the ability to spread easily and affect various tissues in the body, they activate the host’s immune defense, causing the multisystem inflammation and symptoms of the disease [2]. The skin, joints, and neurological system are the most affected by the *Borrelia* infection [3]. Erythema migrans (EM) is the erythematous rash with a centrifugal extension appearing at the site of a tick bite, at the same time being the first sign of the localized *Borrelia* infection [4]. As the spirochetes disseminate through the bloodstream, or tissue planes, different manifestations of LB develop. Lyme neuroborreliosis (LNB) is the early disseminated type of LB, mainly caused by *B. garinii* invasion in the central nervous system (CNS) [3,5]. According to The European Federation of Neurologic Societies (EFNS) guidelines, the diagnosis of definite LNB must be based on the fulfillment of three criteria, and two of them for possible LNB: neurological symptoms, cerebrospinal fluid (CSF) pleocytosis, and Bb-specific antibodies produced intrathecally [6] (Table 1 and Table 2). Neuroborreliosis infection manifests itself by facial nerve palsy, meningitis, and radiculopathy; however, symptoms differ in the European and American population due to different spirochete species [2]. The presence of various clinical features is also related to the age of the patients, as children do not complain of painful meningoradiculitis, indicated as the most common extracutaneous symptom in adults [7].

The following paper presents the summary of the results of the latest research on Lyme neuroborreliosis in children. The collected data were divided into sections, representing various aspects of this disease with emphasis on neurological manifestations, diagnosis, and treatment.

Here are presented the Lyme neuroborreliosis (LNB) definitions according to EFNS guidelines [6] (Table 1 and Table 2).

## 2. Materials and Methods

A search was conducted in the PubMed, Medline and Google Scholar databases to identify the literature related to Lyme neuroborreliosis in children. The following terms were used in the searching process: “Lyme neuroborreliosis”, “children”, “facial palsy”, meningitis”, “symptoms”, “treatment“. Manuscripts were reviewed for titles, abstracts, and the entire text based on the following criteria: (1) original papers; (2) reviews; (3) Lyme neuroborreliosis in children as a key topic of the paper; and (4) Lyme neuroborreliosis in adults, including comparisons to the pediatric population. The exclusion criteria were as follows: (1) methodological studies, editorials, commentaries, letters, and hypotheses; (2) no available abstract; and (3) manuscripts in a language other than English. The analysis was conducted in the following steps. The first step was related to the analysis of selected papers based on titles and abstracts, the second step was connected with the analysis of full-text papers, and the last step included the analysis of the collected data.

## 3. Results

### 3.1. Etiology and Transmission

In Europe, infected Ixodes ricinus is responsible for the transmission of spirochetes and the development of Lyme borreliosis. According to the literature, B. garinii followed by B. afzeli and B. burgdorferi is considered to be the most common etiological agent of neuroborreliosis. However, it needs to be pointed out that the above information applies to adults, as data about the etiological basis of LNB in European children is limited. A study conducted in Slovenia proved to help verify this information, as B. garinii followed by B. afzeli turned out to be dominant in the development of pediatric LNB [8]. 

The data obtained from animal experiments showed that the risk of Borrelia infection is related to the duration of the tick’s blood meal; however, it is not possible to assess the exact time of probable infection, as it varies between the different species [9]. The chances of quick tick removal to decrease the risk of Borrelia transmission often remain small, as the tick attachment to the skin is usually unrecognized by the human. Children are the most exposed to LB infection, as they rarely report the tick bite and are characterized by low adherence to protective measures, such as covering exposed skin and using insect repellents [10]. Observant parents play a significant role in recognizing the bite in locations that are difficult to visualize. Fortunately, according to laboratory tests performed on animals, the transmission of spirochetes within the first 12 hours of tick sucking is rare [2].

### 3.2. Epidemiology

Lyme borreliosis is endemically widespread and its incidence depends on tick abundance and exposure [11]. Subsequently, rural and forested areas are places with a greater risk of acquiring the disease but also urban gardens and parks are places where ticks are commonly found. In Europe, *Borrelia afzelii*, *Borrelia garinii*, *Borrelia bavariensis,* and *B. burgdorferi sensu stricto* (*B. burgdorferi*), and very rarely, *Borrelia spielmanii*, *Borrelia bissettii*, *Borrelia lusitaniae*, and *Borrelia valaisiana* are indicated as etiological factors of LB, while in North America, *Borrelia burgdorferi sensu stricto* remains the almost exclusive cause of this disease [8]. However, referring to this location, it is also worth mentioning the *B. mayonii*, which was recently reported as a novel human pathogenic spirochete causing Lyme disease. *B. miyamotoi* also causes infection, but this is usually more akin to relapsing fever.

According to studies on LNB, it was observed that the anatomical distribution of tick bites was different in children and adults. Among the pediatric population, the most common locations of the tick bites, if successfully reported, are the ears, neck, and especially head (the regions closest to the CNS), which were and still are associated with a high risk of Lyme neuroborreliosis development in children [3].

The highest incidence of LB occurs in the main endemic areas—Scandinavia and Central Europe—and are estimated for more than 300 cases per 100,000 inhabitants [12], while in Western Europe, there are significantly fewer cases reported [13]. Unfortunately, the above information refers to all manifestations of *Borrelia* infections, including neuroborreliosis, which makes the scale of the LNB problem difficult to determine. Additionally, it is not specified to what extent these cases concern children; however, if mentioned, the incidence is defined as rare [14,15,16].

It was estimated that the highest incidence of LB is bimodal and occurs among adults aged 45–59 years and children (with male predominance [11,17,18,19]) aged 5–9 years, with the peak at 7 years [20,21]). According to a Norwegian study, boys with NB were older than girls, their clinical manifestations lasted longer, and higher pleocytosis was observed in the male pediatric population [22].

At present, no figures showing the rate of LB or LNB worldwide occurrence are available, and no population-based studies of the incidence and clinical presentation of serologically confirmed Lyme disease in European children have been carried out [23]. However, it seems that more information will be available in the future, as the EU-wide surveillance for neuroborreliosis has been established [24].

### 3.3. Symptoms

#### 3.3.1. Lyme Neuroborreliosis Symptoms—General Information

Due to the duration of the symptoms, Lyme neuroborreliosis can be divided into the early and the late stage. Each of them are characterized in Table 3 and Table 4.

#### 3.3.2. The Clinical Image in Children with Lyme Neuroborreliosis

Clinical presentation of Lyme neuroborreliosis presents itself differently from weeks to months after exposure. In children, early LNB with a short duration of symptoms is usually distinguished. Small children can additionally present with loss of appetite, fatigue, or changes in mood [6,11,30,31]; however, these symptoms occur along with more typical ones. That is the reason for almost every study to indicate that the occurrence of symptoms should be taken into account with the combination of the epidemiologic situation, endemic areas or season in the year as well as laboratory testing, which can significantly help with the assessment.

**Figure 1:**
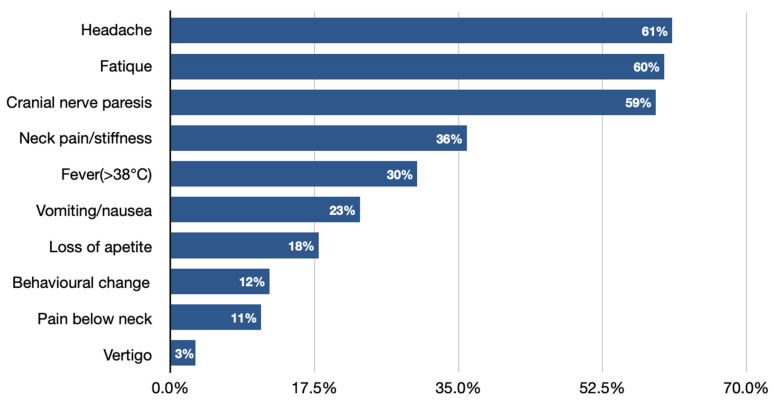
The most common symptoms in children with neuroborreliosis [3].

Facial nerve palsy next to Lymphocytic meningitis is the most common neurological symptom of Lyme neuroborreliosis in the pediatric population (Figure 1) [3,14,32,33,34,35]. It needs to be pointed out that clinical manifestations of LNB, comparing to other CNS infections, significantly differ, as they are often characterized by more insidious onset, cranial nerve palsies (especially facial), and the occurrence of diffuse symptoms, e.g., headache [11,29]. Very rarely, LNB can present with a normal neurological examination (showing no abnormalities in mental status; skull, spine and meninges; cranial nerves; motor examination; sensory examination; coordination; reflexes; and gait and station), which can also lead to misdiagnosis and delay in proper treatment implementation.

#### 3.3.3. Facial Nerve Palsy (FNP)

Facial paralysis as the main symptom should immediately raise suspicion of LNB. It has been already proven that without facial nerve palsy existence and only with the occurrence of diffuse symptoms, the diagnosis is evaluated with a delay. Moreover, FNP is usually a reason for a primary care or pediatric emergency department visit, leading to hospital admittance and indication for further diagnosis. Children are often referred to an ear–nose–throat specialist for otoneurologic examination, which only confirms that the increased awareness of LNB should apply not only to neurologists, but also to doctors of other specialties.

According to the research of Swedish children with LNB in highly endemic areas, facial nerve palsy occurrence was observed in 94% of patients, with only three bilateral FNP cases [3]. That remains consistent with other studies, stating unilateral facial nerve palsy as the most common type in children [36]. Abducens nerve affection was reported in 3% of all cases. The trigeminal nerve was affected in two children and the trochlear nerve in one patient. Additionally, the authors mention transient hemiparesis occurrence among their patients as well as nonfebrile, generalized tonic–clonic seizures, which, according to the literature, co-exist with parenchymatous lesions or meningoencephalitis. Other studies also confirmed the high FNP incidence in children with LNB. The English study showed the results of 84% FNP cases [36]. What is interesting is that the Norwegian research on 142 children indicated FNP occurrence to be more common among girls (86%) than boys (62%). There were also differences in other symptoms depending on gender. For example, headache and/or neck stiffness occurred in boys more frequently (30%) than in girls (10%) [18]. In contrast to these results, the opposite data were published by German research, stating that 11 of 16 children with FNP were male [33].

#### 3.3.4. Severity and Duration of FNP

A study of 98 children with LNB presenting with facial nerve palsy at admission indicated that the severity of facial nerve dysfunction (The House Brackman grading system) was described most frequently as moderate (19%), and severe (12%), while mild (5%), and total loss of FNP function (8%) were lower [11]. The most common related clinical features were as follows: headache or meningeal symptoms (60%), EM/lymphocytoma (16%), and vertigo/nystagmus (13%). The same study (*n* = 177 total cases) reported 6% patients at the 2-month follow-up having persistent symptoms after facial nerve palsy: eye-closing impairment (*n* = 1), affected pronunciation (*n* = 2), extensive tear secretion (*n* = 2), and social consequences (*n* = 1). However, the cosmetic problem (*n* = 5) was indicated as the most common, affecting the patients’ quality of life.

#### 3.3.5. Lumbar Puncture

Formal recommendations regarding lumbar puncture (LP) in children still remain challenging. However, in most cases of neuroborreliosis, this diagnostic procedure is performed to exclude other diagnoses [37]. This is usually a matter of agreement, and recommendations are published in local guidelines of endemic areas, such as in Stockholm, where the routine procedure of children with FNP consists of ward admittance and general examination, cranial neurological examination, and blood tests as well as lumbar puncture [38]. The majority of single-center guidelines recommend LP in any case of LNB suspicion, even if there are no meningeal symptoms and other symptoms characteristic of neuroinfections in a patient with peripheral paralysis or without paresis of the facial nerve. The most common justification of the need for LP performance is the detection of pleocytosis, which is one of the three criteria for the diagnosis of definite neuroborreliosis, according to the EFNS criteria [6]. However, some authors consider lumbar puncture as controversial, indicating that the obtained results have no impact on further clinical management.

#### 3.3.6. Differential Diagnosis of Facial Nerve Palsy

As facial nerve palsy is always an alarming sign, the cause of its occurrence should always be carefully investigated. The onset, time course, eventual progression, and concomitant diseases are the most important factors allowing an assessment of the etiology of FNP [39,40,41,42,43]. The possible causes of acquired facial nerve palsy in children, facilitating the differential diagnosis, are presented in Table 5 [44,45,46]. It should be remembered that the type of treatment depends on the correct diagnosis of the paralysis cause and its severity, and thus requires the cooperation of a multidisciplinary team of specialists.

#### 3.3.7. The Occurrence of Erythema Migrans in Children with Lyme Neuroborreliosis

It was proven that children with Erythema migrans occurrence in the head and neck area more frequently presented with facial nerve palsy (36%) and in 94% of cases, it was an ipsilateral type of FNP [47]. It was also indicated that children with EM in the head and neck area presented with ipsilateral facial nerve palsy in 94% of cases. Additionally, the majority of patients with EM had the diagnosis of LNB evaluated in a shorter period of time.

#### 3.3.8. Lyme Meningitis

Lyme meningitis in children with neuroborreliosis can be easily overlooked due to very mild symptoms. However, the course of the disease is different when cranial nerve deficits occur.

The data collected indicate isolated meningitis (without radicular symptoms) as most common among children [14,25,28,34]. In every case of LNB, the following changes in CSF can be observed: pleocytosis, disruption of the blood–CSF barrier, and intrathecal immunoglobulin synthesis [2]. However, in the very early stages of LNB, the CSF parameters can be normal [48]. The research of Stanek G. et al. shows that pleocytosis with WBC ≥ 7 and > 90% mononuclear cells in CSF are features characteristic of pediatric neuroborreliosis [12].

Lyme meningitis can overlap with the viral one, but it is possible to distinguish them, as symptoms of LNB are of less acute onset. Further research showed that neuroborreliosis was the most common causative agent of meningitis in children aged 5–9 years, while *Haemophilus influenzae* type b, pneumococci, and meningococci caused much lower incidence [3]. That fact should raise awareness and support the consideration of Lyme neuroborreliosis as a childhood disease. 

#### 3.3.9. The Comparison of the Clinical Image Caused by *Borrelia garinii* and *Borrelia afzelii*

**Figure 2:**
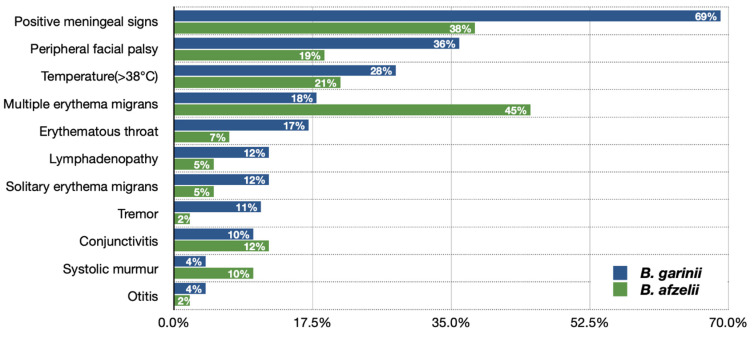
Presentation of the symptoms in children with Lyme neuroborreliosis caused by *Borrelia garinii* and *Borrelia afzelii* [36].

The frequency of various clinical presentations in children with Lyme neuroborreliosis depends on the etiological agent and is different when caused by *Borrelia garinii* and *Borrelia afzelii* (Figure 2) [36]. The original study conducted in Slovenia reported significant insights of *B. garinii* clinical image being more often not suggestive of CNS involvement but more pronounced CNS inflammation than in *B. afzelii* infection [8].

### 3.4. Diagnostic Process

When assessing patients for Lyme neuroborreliosis, the following factors should be taken into consideration: specific clinical symptoms of LNB, the likelihood of the patient’s exposure to infected ticks, the possibility of other illnesses with similar symptoms, and lastly, the results of serological and sometimes other diagnostic tests. In many cases, the currently used laboratory techniques have only limited possibilities to differentiate between previous and active *Borrelia* infection. The history and physical examination are, therefore, of decisive importance.

#### 3.4.1. The Laboratory Diagnostics of CSF

A lumbar puncture is performed in the majority of cases when NB in a child is suspected. The limitation of the LP procedure is associated with the lack of parental consent, not rarely noted in the literature. Inflammatory CSF changes include pleocytosis with the lymphocytes predominance, blood–CSF barrier dysfunction and intrathecal immunoglobulin synthesis, with the exception in the very early stage [2]. Attention should be also paid to elevated protein levels, while glucose is within the normal range [11]. Normal CSF cytosis in children should prompt repeated CSF examination. The early stage of the disease among immunocompromised patients or NB caused by the etiological agent of *B. afzelii* are considered to be the reasons for such normal CSF results [49].

It is also worth paying attention to the research of Rožič, Mojca MD et al. who showed CSF findings in Slovenian children according to different etiological agents causing LNB [8].

#### 3.4.2. Intrathecal Antibody Production

When the serological examination for Lyme disease is positive or when there is a strong suspicion of neuroborreliosis in the absence of antibodies in the blood, the cerebrospinal fluid is examined, and intrathecal (non-*Borrelia*-specific IgM) antibody production is assessed. The detection of intrathecal synthesis of antibodies against *B. burgdorferi*, indicated as a gold standard, confirms the diagnosis of definite LNB [29]. For the determination of intrathecal antibody production, a sample for the determination of antibodies in the blood on the day of CSF collection is taken. The diagnostic sensitivity is estimated in approximately 80% of children presenting with clinical manifestations of LNB for less than 6 weeks and increases with the duration of the symptoms [50]. The negative results can be obtained in 20–25% of patients [18,51].

#### 3.4.3. Antibodies in the Serum

ELISA (screening test) and Western blot (confirmation test) are the two-stage basic diagnostic procedures used for the detection of antibodies in the serum [52]. When there is a negative serum serological test result and the LNB is still considered a potential cause of clinical manifestations, the antibody testing should be done 2–4 weeks later in anticipation of seroconversion.

#### 3.4.4. Relevant *Borrelia* Antigens

There is a large number of *B. burgdorferi* diagnostically relevant antigens (Table 6), which depend on the stage of the disease. The immunoblot technique allows to separate them, and the knowledge about their occurrence helps to interpret correctly the serological test results.

#### 3.4.5. *B. burgdorferi* Bacterial Culture Growth

*Borrelia* cultivation from CSF is not recommended and not widely used as a routine procedure, due to many culturing requirements. The bacteria need to be grown in specific liquid culture media or on the Barbour–Stoenner–Kelly (BSK) medium at 30–34 °C but its sensitivity is suboptimal, ranging from 10% to 30%, which limits its use in clinical practice [6,52,57]. Additionally, the culturing is a time-consuming (2–3 months) [33] process, and there is a need for specialized microbiological laboratories [6].

#### 3.4.6. Polymerase Chain Reaction (PCR)

Polymerase chain reaction (PCR) may be used for detection of the *Borrelia* genome in cerebrospinal fluid (CFS) to aid in the diagnosis of recent neuroborreliosis with a short duration of neurological symptoms [58].

Due to the small number of copies of the spirochete in samples, the sensitivity of the PCR-based test is low with a sensitivity of 5% [59], and can be even lower in the case of late LNB, as spirochetes easily migrate to various CNS tissues [6,60]; therefore, a negative PCR result does not exclude Lyme neuroborreliosis. For this reason, the PCR test alone is not definitive in the diagnosis of LN disease. Determination of antibody synthesis is always a more sensitive alternative in late neuroborreliosis and is usually more sensitive in early neuroborreliosis as well. The specificity of this detection technique is theoretically close to 100% [59] and therefore, the detection of *Borrelia* DNA in CSF is evidence of Lyme disease.

#### 3.4.7. Chemokine CXCL13

Determination of CXCL13 in CSF appears to be a promising additional parameter for the diagnosis of early neuroborreliosis in addition to antibody determination, especially in cases where the antibody response is still negative and general parameters, such as pleocytosis and the albumin ratio, provide insufficient definitions for the diagnosis of neuroborreliosis [49,61,62]. The diagnostic sensitivity for CXCL13 is estimated at 88% and specifically at 89% [63,64]. However, there are also significantly lower values of sensitivity of CXCL13 in CSF and are pre-treated with antibiotics before CSF sampling [62]. The studies have mainly examined patients with possible neuroborreliosis for which an increased CXCL13 level was found in 73% of cases [5]. It is not yet clear whether the parameter also has value in late neuroborreliosis. Of great importance remains the fact that after the administration of antibiotic therapy, the chemokine level drops, which is suggestive of its significant role as a disease activity and treatment effectiveness marker [49,64,65]. However, CXCL13 cannot be used as a predictive marker for recovery, as research has shown its low concentration on admission among children, who later reported persistent symptom occurrence at the 2-month follow-up [5]. Although chemokine is considered a useful marker, the current official recommendations do not include its labeling as a routine procedure in the LNB diagnostic process, as there is still a lack of sufficient research.

#### 3.4.8. Magnetic Resonance Imaging (MRI)

In patients with neuroborreliosis, imaging such as CT scans and MRI of the brain or spinal cord may show focal abnormalities. The comparable abnormalities, such as enhancement of meninges, enhancement of cranial nerves, vasculitis, ischemic foci, or diffuse CNS parenchymal involvement, are also found in other conditions, so the imaging techniques mentioned have no diagnostic value in establishing a diagnosis of Lyme neuroborreliosis [37]. However, clinicians must heighten their awareness of pseudotumor-like picture occurrence in MRI, including visual obscurations and visual loss among children with Lyme meningitis [66].

### 3.5. Treatment

Neuroborreliosis is a disease that can be effectively treated by antibiotics. However, the implementation of the medications must be preceded by an LNB diagnosis, which in the pediatric population is often delayed or overlooked due to non-specific symptom occurrence. Based on numerous publications, data collected on the treatment of Lyme neuroborreliosis in children are still insufficient or of low quality. Moreover, studies focused on that aspect were conducted even several decades ago, which cannot be accepted in relation to the current clinical research standards. 

Despite the existence of many different recommendations on how to treat neuroborreliosis in children, the most important factor is an individual approach to the patient; however, each treatment regimen should be based on clinical evidence. The clinician’s attention must be paid to the patient’s age, allergies, pregnancy, tolerability or other diseases occurrence, etc., to choose the most effective treatment option. The current recommendations indicate that one of the following medications should be implemented to manage both early and late Lyme neuroborreliosis: penicillin G, ceftriaxone, cefotaxime, or doxycycline. However, a dose of antibiotics, the frequency of its application, route of administration, and duration of treatment remain under discussion. The last of the mentioned factors seems to be particularly important nowadays, as unreasonable extended antibiotic courses may result in a growing problem of multidrug-resistant bacteria.

#### 3.5.1. The Antibiotic Treatment Recommendations of Lyme Neuroborreliosis

Although there are different guidelines for treating Lyme neuroborreliosis (Table 7, Table 8, Table 9 and Table 10), all recommendations are based on the use of antibiotics characterized by good penetration into the CNS. The treatment should be initiated in all patients with neurological manifestations typical of LNB, inflammatory CSF changes, and positive *Borrelia* serology, while in cases of possible LNB, antibiotics can only be considered after a differential diagnosis, excluding other diseases.

#### 3.5.2. Restrictions on the Use of Doxycycline

The implementation of doxycycline remains contraindicated in children under 8 years, as it may cause teeth staining and enamel hypoplasia due to incomplete dental enamel formation. However, some data indicate that this undesirable effect can be prevented by avoiding sunlight. Some authors even put forward the conclusion that the risk of side effects appearance after short-term use of doxycycline remains minimal, and the potential benefits of using this drug outweigh this risk [67]. Nevertheless, a different alternative antibiotic should be chosen in this age group.

#### 3.5.3. The Antibiotics Treatment Side Effects

All the antibiotics recommended for the treatment of LNB are considered effective and safe, and serious side effects were reported very rarely. Table 11 presents the possible side effects and was created only based on very limited data, but it may raise the awareness of doctors implementing the treatment to their patients.

#### 3.5.4. Non-Antibiotic Treatment of Lyme Neuroborreliosis

According to different studies, steroids are listed among the medications used to treat Lyme disease [70,71,72]; however, the current data about steroids implementation with antibiotics or itself are still limited, especially in the pediatric group. The use of steroids requires special attention in patients with peripheral paralysis of nerve VII. As this controversial way of treatment is still under debate among clinicians and there is a lack of randomized controlled trials, steroids remain not recommended. The authors also emphasize the very high rate (95%) of spontaneous remission in children, which also speaks in favor of not using glucocorticoid therapy in the pediatric group of patients.

#### 3.5.5. Treatment Monitoring and Clinical Outcome

The effectiveness of the treatment can be assessed by the clinical manifestations.

Pediatric patients presenting with facial nerve palsy caused by neuroborreliosis recovered faster than those with FNF of different etiology; moreover, the recovery time was observed to be shorter among younger children (aged 0–8 years). It was also stated that after 12 weeks, the overall complete recovery was observed in more than 97% of cases and the rate of recovery did not depend on etiology or age.

The study of 618 children with Lyme neuroborreliosis showed complete recovery in 29% of the patients by the end of the treatment, and in 63% after 4–6 weeks [3].

In general, the clinical outcome is significantly better in children than in adults. Among the pediatric population, the neuropsychological prognosis is favorable and long-term neuropsychologic disorders are not seen in the pediatric population, compared to adults struggling with cognitive disorders and persistent or recurrent neurologic symptoms [73,74,75]. However, one-fifth of children with LNB suffer from sensory or motor sequelae [17]. Moreover, in 11/16 of these children, sequelae were the residua of facial palsy. In the other five, these were residua of other, more severe deficits in acute disease. Persistent dysfunction after facial nerve palsy occurs in 18–22% of patients [76,77]. The majority of patients, both at discharge from hospital (29%) or after 4–6 weeks follow-up (63%) reported full recovery.

## 4. Conclusions

Lyme neuroborreliosis in children is considered an uncommon disease, but many reasons may hide the true scale of this problem. The lack of registration of incidence among children, particularly in areas according to specific criteria, in the past and the lack of population-based studies among children make neuroborreliosis disease an excellent field for research.

Undeniably, the diagnosis of Lyme disease among children is challenging, requiring great knowledge and perceptiveness from clinicians. Due to the multitude of non-specific symptoms, it is easy to misdiagnose and delay the proper treatment. Statistically, most children with symptoms suggesting neuroborreliosis are admitted to primary health care and the general practitioner plays a key role in the diagnosis of a patient with Lyme disease [36]. Of great importance is also increasing the awareness of all health professionals, which can result in fast diagnosis and implementation of appropriate treatment.

It needs to be pointed out that due to global warming, the incidence of LNB will be ever increasing, posing a serious public health problem. There are several steps to achieve better disease control. It is, therefore, extremely important to educate the general society about preventive strategies and alarming symptoms, especially in endemic areas [78].

## Figures and Tables

**Table 1:** Criteria for definite and possible Lyme neuroborreliosis (LNB) [6].

Definite neuroborreliosis * - fulfillment of 3 criteria	Neurological symptoms suggestive of LNB *** Cerebrospinal fluid (CSF) pleocytosis Intrathecal *Borrelia burgdorferi sensu lat*o (Bb) antibody production
Possible neuroborreliosis - fulfillment of 2 criteria **	

* All subclasses of LNB except for late LNB with polyneuropathy. ** If criteria 3 is lacking, Bb-specific IgG antibodies in the serum have to be found after a duration of 6 weeks. *** All other causes must be excluded.

**Table 2:** Criteria for definite late Lyme neuroborreliosis (LNB) with polyneuropathy [6].

Peripheral neuropathy
Clinical diagnosis of acrodermatitis chronica atrophicans (ACA)
*Borrelia burgdorferi sensu lato* (Bb)-Specific antibodies in serum

**Table 3:** Lyme neuroborreliosis classification [6].

Early LNB	Late LNB
Neurological symptoms existing for <6 months	Neurological symptoms existing for >6 months
PNS manifestations (Bannwarth’s syndrome, other peripheral neurological manifestations are plexus neuritis and mononeuritis multiplex	PNS manifestations (mononeuropathy, radiculopathy and polyneuropathy)
CNS manifestations (confusion, cerebellar ataxia, opsoclonus–myoclonus, ocular flutter, apraxia, hemiparesis or Parkinson-like symptoms)	CNS manifestations (cerebral vasculitis, chronic progressive Lyme encephalitis or encephalomyelitis with tetraspastic syndrome, spastic–ataxic gait disorder and disturbed micturition)

Abbreviations: PNS—peripheral nervous system, CNS—central nervous system.

**Table 4:** General information on the clinical course of early and late neuroborreliosis [2,25,26,27,28,29].

Lyme Neuroborreliosis Stage	Early Lyme Neuroborreliosis	Late Lyme Neuroborreliosis (Chronic Lyme Neuroborreliosis)
Persistence of symptoms	weeks to months [25,26,27]	months to years [25,26,27]
Occurrence	over 91% of cases—patients with neuroborreliosis having early manifestations (a total number of 330 patients) [26]	less than 9% of cases—patients with neuroborreliosis having late manifestations (a total number of 330 patients) [26]
Neurological symptoms appearance	painful meningopolyradiculitis, unilateral or bilateral facial paresis (Bannwarth’s syndrome), cranial neuritis, plexus neuritis, mononeuritis multiplex meningitis in children	encephalomyelitis with spastic atactic gait disturbance bladder dysfunction Isolated meningitis is very rare
Characteristic trait/distinguishing feature	radicular pain	rarely any pain

**Table 5:** Possible causes of acquired facial nerve palsy in children [44,45,46].

Etiology	Infectious	Inflammatory	Neoplastic	Traumatic
**Symptoms**	Ramsay Hunt Syndrome Epsterin–Barr virus *Haemophilus influenzae* Tuberculosis Lyme disease Cytomegalovirus Adenovirus Rubella Mumps *Mycoplasma pneumonia* Human immunodeficiency virus Acute otitis media	Henoch–Schonlein porpora Kawasaki syndrome	Schwannomas of the VII c.n. Hemangiomas Rhabdomyosarcoma Temporal bone histiocytosis Leukemia Parotid gland tumors	Temporal bone fracture Iatrogenic

**Table 6:** Immunologically relevant *Borrelia* antigens [2,53].

Early Immune Response (Mainly IgM) [54,55,56]	Late Immune Response (Mainly IgG) [55]
Flagellar protein, OspC, VlsE	P83/100, p58, p43, p39, p30, p21, DbpA (Osp17), p14, VlsE

**Table 7:** Overview of antibiotic treatment according to guidelines for diagnosis and treatment in neurology—Lyme neuroborreliosis [68].

Antibiotic	Pediatric Dose (Dose/kg × Day)	Duration (Days)
**Early Lyme neuroborreliosis**		
Ceftriaxone	50 mg	14
Cefatoxime	100 mg	14
Penicillin-G	200–500,000 IU	14
Doxycycline	Age 9 and up, 4 mg (maximum 200 mg)	14
**Late Lyme neuroborreliosis**		
Ceftriaxone	50 mg	14–21
Cefatoxime	100 mg	14–21
Penicillin-G	200–500,000 IU	14–21
Doxycycline	Age 9 and up, 4 mg (maximum 200 mg)	14–21

**Table 8:** Overview of antibiotic treatment according to National Institute for Health and Care Excellence (NICE) guideline for children aged 9–12 [69].

Children Aged 9–12		
Antibiotics	Dosage	Duration (Days)
**Lyme disease affecting the cranial nerves or peripheral nervous system**		
Doxycycline (oral) (children under 45kg)	5 mg/kg in 2 divided doses on day 1 followed by 2.5 mg/kg daily in 1 or 2 divided doses For severe infections, up to 5 mg/kg daily	21
Amoxicillin (oral) (children under 33 kg)	30 mg/kg 3 times per day	21
**Lyme disease affecting the central nervous system**		
Ceftriaxone (intravenous) (children under 50 kg)	80 mg/kg (up to 4 g) once per day	21
Doxycycline (oral) (children under 45 kg)	5 mg/kg in 2 divided doses on day 1 followed by 2.5 mg/kg daily in 1 or 2 divided doses For severe infections, up to 5 mg/kg daily	21
**Lyme disease arthritis and acrodermatitis chronica atrophicans**		
Doxycycline (oral) (children under 45 kg)	5 mg/kg in 2 divided doses on day 1 followed by 2.5 mg/kg daily in 1 or 2 divided doses For severe infections, up to 5 mg/kg daily	28
Amoxicillin (oral) (children under 33 kg)	30 mg/kg 3 times per day	28
Ceftriaxone (intravenous) (children under 50 kg)	80 mg/kg (up to 2 g) once per day	28
**Lyme carditis B**		
Doxycycline (oral) (children under 45 kg)	5 mg/kg in 2 divided doses on day 1 followed by 2.5 mg/kg daily in 1 or 2 divided doses	21
Ceftriaxone (intravenous) (children under 50 kg)	80 mg/kg (up to 2 g) once per day	21

B For hemodynamically unstable Lyme carditis, intravenous ceftraxione should be used as first choice.

**Table 9:** Overview of antibiotic treatment according to National Institute for Health and Care Excellence (NICE) guideline for children under 9 years [69].

Children Under 9 Years		
Antibiotics	Dosage	Duration (Days)
**Lyme disease affecting the cranial nerves or peripheral nervous system**		
Amoxicillin (oral) (children under 33 kg)	30 mg/kg 3 times per day	21
**Lyme disease affecting the central nervous system**		
Ceftriaxone (intravenous) (children under 50 kg)	80 mg/kg (up to 4 g) once per day	21
**Lyme disease arthritis and acrodermatitis chronica atrophicans**		
Amoxicillin (oral) (children under 33 kg)	30 mg/kg 3 times per day	28
Ceftriaxone (intravenous) (children under 50 kg)	80 mg/kg (up to 2 g) once per day	28
**Lyme carditis (both hemodynamically stable and unstable)**		
Ceftriaxone (intravenous) (children under 50 kg)	80 mg/kg (up to 2 g) once per day	21

**Table 10:** Overview of antibiotic treatment according to National Institute for Health and Care Excellence (NICE) guideline for children aged 12 and over [69].

Children Aged 12 and Over		
Antibiotics	Dosage	Duration (Days)
**Lyme disease affecting the cranial nerves or peripheral nervous system**		
Doxycycline (oral)	100 mg twice per day or 200 mg once per day	21
Amoxicillin (oral)	1 g 3 times per day	21
**Lyme disease affecting the central nervous system**		
Ceftriaxone (intravenous) A	2 g twice per day or 4 g once per day	21
Doxycycline (oral)	200 mg twice per day or 400 mg once per day	21
**Lyme disease affecting the central nervous system**		
Doxycycline (oral)	100 mg twice per day or 200 mg once per day	28
Amoxicillin (oral)	1 g 3 times per day	28
Ceftriaxone (intravenous)	2 g once per day	28
**Lyme carditis B**		
Doxycycline (oral)	100 mg twice per day or 200 mg once per day	21
Ceftriaxone (intravenous) C	2 g once per day	21

A When an oral switch is being considered, use doxycycline; B Hemodynamically unstable; C Do not use azithromycin to treat people with cardiac abnormalities associated with Lyme disease because of its effect on QT interval.

**Table 11:** Unfavorable clinical courses under various antibiotics treatment [2,3,69].

Antibiotic	Side Effect
Doxocycline (in children under 8 years)	Teeth staining and enamel hypoplasia
	Vomiting
Penicillin G	Moderate allergic skin reaction
	Increase in liver enzymes
Ceftriaxone	Asymptomatic gallbladder concrements
Azithromycin	QT interval prolongation
Any	Jarisch–Herxheimer reaction
